# Rhenium Biscorrole Sandwich Compounds: XAS Evidence for a New Coordination Motif

**DOI:** 10.1021/acs.inorgchem.3c00632

**Published:** 2023-05-23

**Authors:** Abraham B. Alemayehu, Macon Jedediah Abernathy, Jeanet Conradie, Ritimukta Sarangi, Abhik Ghosh

**Affiliations:** † Department of Chemistry, University of Tromsø, N-9037 Tromsø, Norway; ‡ Stanford Synchrotron Radiation Lightsource (SSRL), SLAC National Accelerator Laboratory, Stanford University, Menlo Park, California 94025, United States; § Department of Chemistry, University of the Free State, P.O. Box 339, Bloemfontein 9300, Republic of South Africa

## Abstract

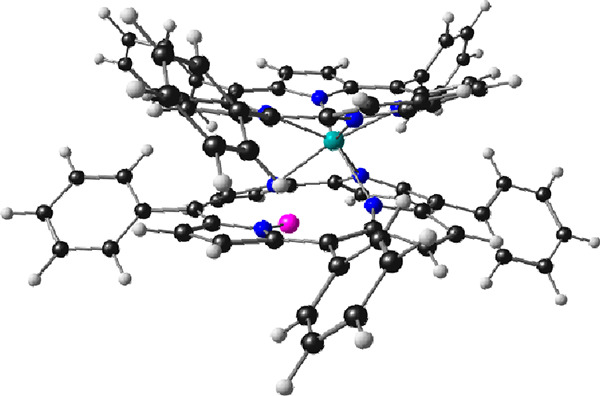

The interaction of three free-base *meso*-tris(*p*-X-phenyl)corroles H_3_[T*p*XPC] (X = H, CH_3_, OCH_3_) with Re_2_(CO)_10_ at 235 °C in the presence of K_2_CO_3_ in *o*-dichlorobenzene has led to putative rhenium biscorrole sandwich compounds with the formula ReH[T*p*XPC]_2_. Density functional theory calculations and Re L_3_-edge extended X-ray absorption fine structure measurements suggest a seven-coordinate metal center, with the “extra” hydrogen located on one of the corrole nitrogens. The complexes can be deprotonated by a base such as 1,8-diazabicyclo[5.4.0]undec-7-ene, resulting in a substantial sharpening of the UV–vis spectra and split Soret bands, consistent with the generation of *C*_2_-symmetric anions. Both the seven-coordinate neutral and eight-coordinate anionic forms of the complexes represent a new coordination motif in the field of rhenium–porphyrinoid interactions.

## Abstract 2

The high-temperature interaction of free-base *meso*-triarylcorroles with Re_2_(CO)_10_ has led to putative rhenium biscorrole sandwich compounds with the formula ReH[Cor]_2_ (Cor = corrole). Density functional theory calculations and Re L_3_-edge extended X-ray absorption fine structure measurements suggest a seven-coordinate metal center, with the “extra” hydrogen residing on one of the corrole nitrogens.

The interaction of rhenium with porphyrin-type ligands has resulted in a growing variety of coordination motifs in recent years.^1,2^ Thus, porphyrins^3^ and related ligands (such as sapphyrin^4,5^ and triphyrin^6^) act as tridentate ligands toward the [Re(CO)_3_]^+^ fragment to yield unusual “capped” organometallic complexes that obey the 18-electron rule. Likewise, ^99^Tc(CO)_3_-capped porphyrin derivatives are also well-known.^7^ Pentavalent rhenium oxo^8^ and rhenium nitrido^9^ porphyrins have also been known for decades. The next major developments came in the 1980s in the form of monomeric rhenium(II) porphyrins^10^ and metal–metal triple-bonded rhenium(II) porphyrin dimers.^11,12^ Rhenium corroles are of more recent provenance: although one was serendipitously isolated some time ago,^13^ a general synthetic route to Re^V^O corroles^14^ (as well as to ^99^TcO corroles^15^) emerged only a few years ago.^16^ The simplicity of latter route has also allowed for peripheral functionalization of Re^V^O corroles via electrophilic aromatic substitution reactions such as halogenation^17^ and formylation.^18^ Another exciting, recent development has been the synthesis of metal–metal quadruple-bonded rhenium corrole dimers.^19^ Detailed electrochemical^20^ and density functional theory (DFT)^21^ studies of these complexes have yielded a host of new insights into metal–metal quadruple bonding. Herein we provide strong spectroscopic evidence for yet another coordination motif for rhenium in the form of rhenium biscorrole sandwich compounds with the molecular formula ReH[T*p*XPC]_2_, where T*p*XPC denotes a generic *meso*-tris(*p*-X-phenyl)corrole. One of the corroles in these complexes is thought to be monoprotonated, which results in a seven-coordinate rhenium(V) center.

Rhenium biscorrole sandwich compounds, ReH[T*p*XPC]_2_, were serendipitously discovered as we attempted to optimize the current synthetic protocol for metal–metal quadruple-bonded rhenium corrole dimers.^19^ Heating Re_2_(CO)_10_ and free-base corroles H_3_[T*p*XPC] (X = H, CH_3_, OCH_3_) in the presence of a base in 1,2-dichlorobenzene at 150–190 °C resulted in the exclusive formation of Re^V^[T*p*XPC](O).^14^ Increasing the temperature to 190–220 °C resulted in the formation both Re^V^O corroles and metal–metal quadruple-bonded rhenium corrole dimers {Re[T*p*XPC]}_2_.^19^ Given the clear role of temperature in determining the product profile, we reasoned that the use of even higher temperatures might lead to higher yields of rhenium corrole dimers. Increasing the temperature to 235 °C (*Caution!*), to our surprise, led to yet a third product in addition to Re^V^O corrole and the rhenium corrole dimer. High-resolution electrospray ionization mass spectrometry (HR-ESI-MS) in the positive mode indicated the molecular formula ReH[T*p*XPC]_2_ for the new products, which, gratifyingly, could be isolated for each of the three corrole ligands examined (Figure 1).

**Figure 1:**
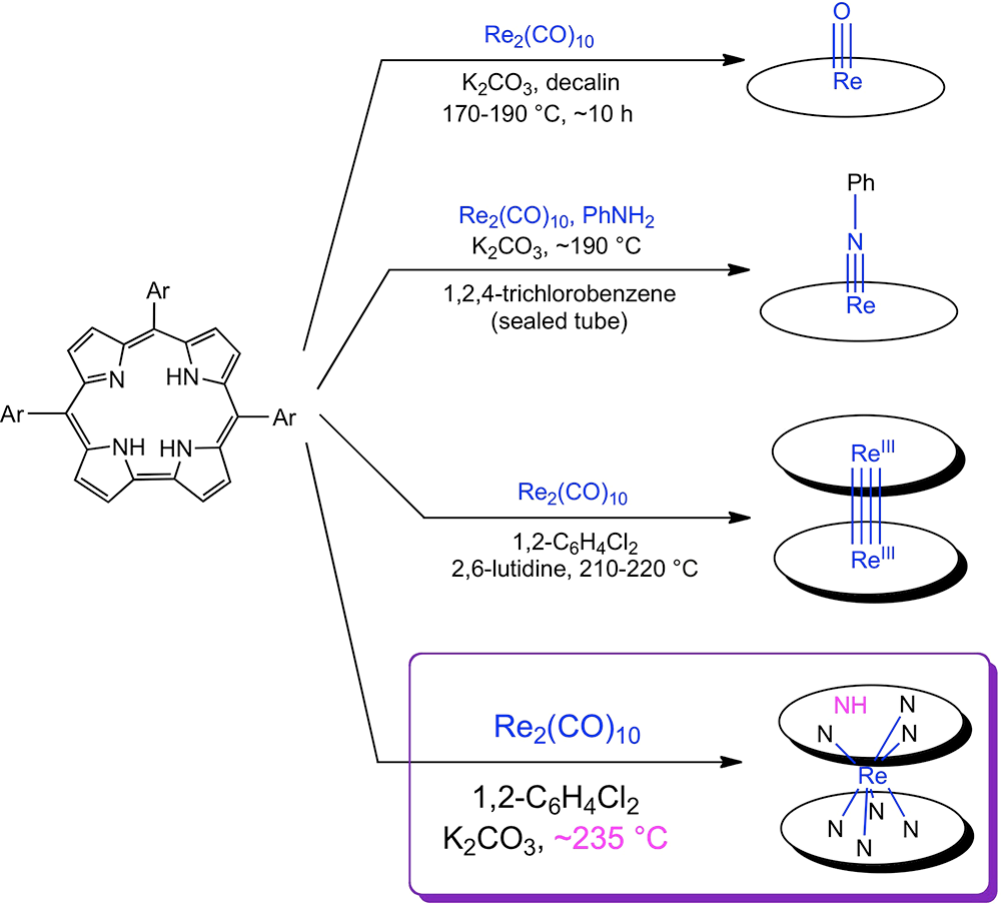
Summary of the interactions between rhenium and corroles as currently elucidated. Inset: this work.

As for molybdenum and tungsten biscorrole complexes,^22−24^ the exceedingly crowded ^1^H NMR spectra of the new compounds, aside from confirming their diamagnetic nature, proved indecipherable. No hydridic protons were observed, and the NH proton signals in general were also found to be broad and not readily discernible. For one compound, ReH[T*p*OCH_3_PC]_2_, natural abundance ^15^N–^1^H heteronuclear single quantum coherence did lead to plausible identification of the NH proton to a broad singlet at −0.64 ppm. All three complexes also failed to yield single-crystal X-ray structures. Fortunately, mass spectrometry, optical and X-ray absorption (XAS) spectroscopies, and DFT modeling studies^25−27^ paint a fair picture of the structure and nature of the compounds. As for molybdenum and tungsten biscorroles,^22,23^ the Soret maxima of ReH[T*p*XPC]_2_ in dichloromethane and toluene proved to be significantly blue-shifted relative to those of free-base corroles, indirectly lending support to the sandwich formulation (Figure 2). Unusually for electronically innocent metallocorroles,^16^ the Soret maxima were found to exhibit significant redshifts with increasing electron-donating character of the substituent X, a behavior typically observed for noninnocent metallocorroles.^27,28^ We tentatively ascribe this observation to the unusual geometry of the complex and to charge-transfer character involving the empty Re 5d_*z*^2^_ orbital. Upon stirring with 1,8-diazabicyclo[5.4.0]undec-7-ene in anhydrous toluene, the UV–vis spectra sharpened dramatically, consistent with the formation of a *C*_2_-symmetric {Re[T*p*XPC]_2_}^−^ anion. The UV–vis spectra of the putative anion are characterized by a deeply split Soret band, with the main feature at ∼438 nm and a prominent, left shoulder at ∼368 nm for X = H and CH_3_ and at ∼379 nm for X = OCH_3_, again reflecting a significant substituent effect. Importantly, HR-ESI-MS analysis of the putative anions (in methanolic solution) in the negative mode revealed a molecular ion with a mass 1 Da lower than that observed for the neutral compounds (see the Supporting Information).

**Figure 2:**
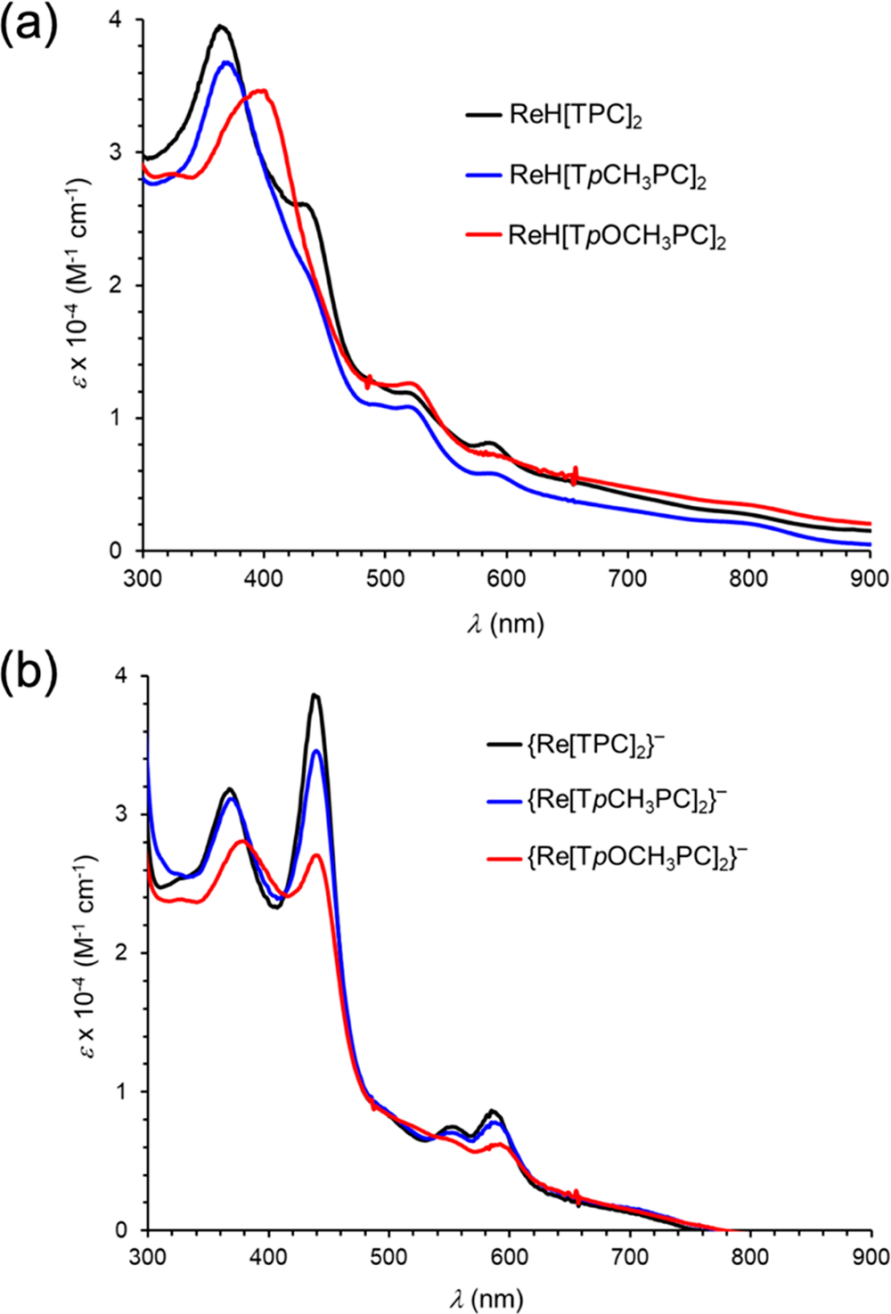
UV–vis spectra of (a) ReH[T*p*XPC]_2_ and (b) putative {Re[T*p*XPC]_2_}^−^ (X = H, CH_3_, and OCH_3_) anions in anhydrous toluene.

To determine the most probable location of the “extra” hydrogen in the neutral sandwich compounds, all-electron scalar-relativistic DFT (OLYP^29,30^-D3^31^/ZORA-STO-TZ2P, as implemented in the ADF program system^32^) geometry optimizations were carried out on a variety of potential tautomeric forms of ReH[TPC]_2_. Assuming an approximate square antiprism of the corrole nitrogens (with the corrole rings rotated approximately 135° relative to each other^22,23^), the global minimum appears to be an nitrogen-protonated tautomer, where the protonated nitrogen belongs to one of the pyrrole rings distal with respect to the direct pyrrole–pyrrole linkage (Figure 3). A rhenium-protonated form could not be located as a local minimum because it spontaneously evolved to the global minimum over the course of the geometry optimization. *meso*-Carbon-protonated forms were also found to be >1.5 eV above the global minimum and so were not considered realistic contenders for the actual structure.

**Figure 3:**
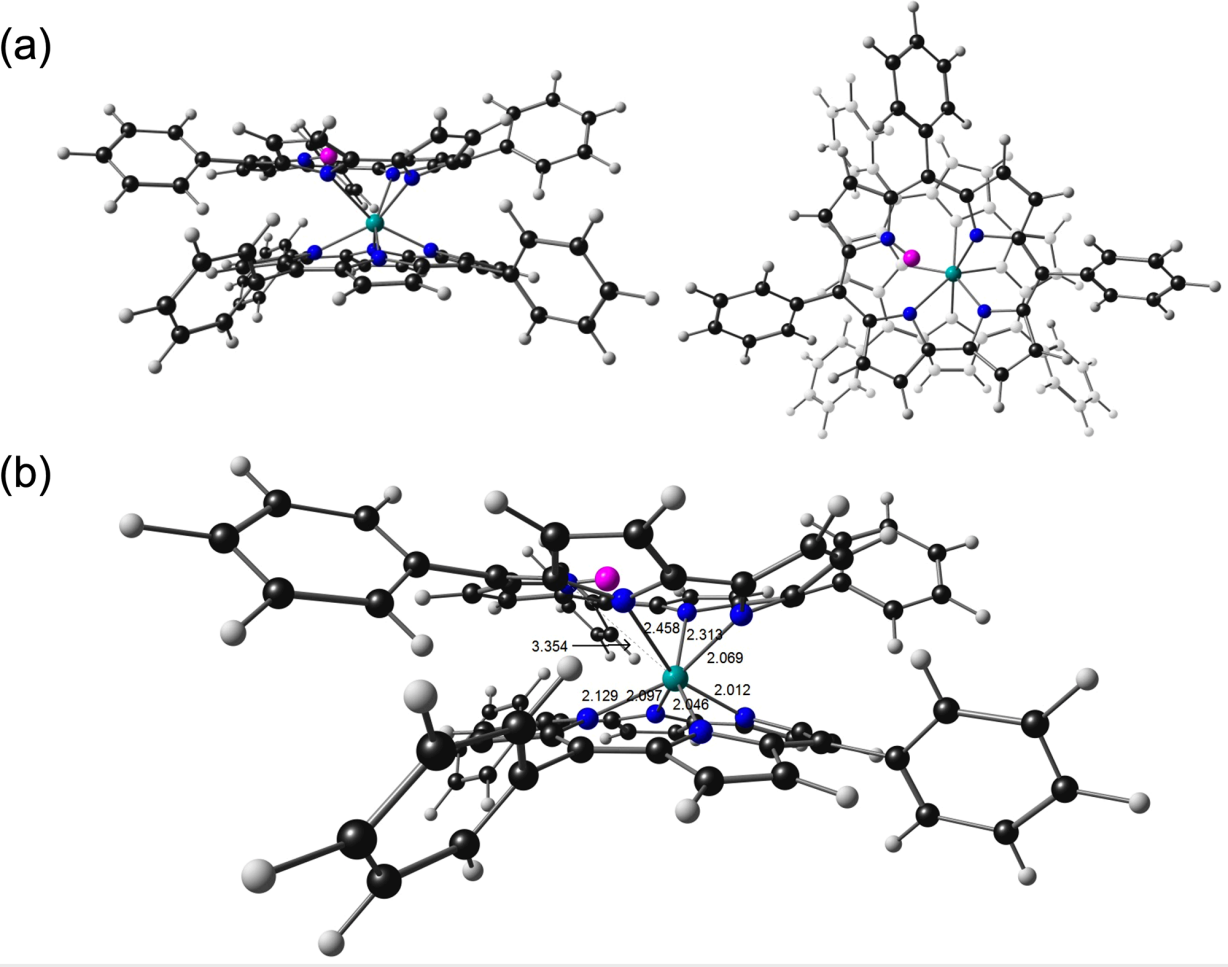
All-electron OLYP-D3/ZORA-STO-TZ2P-optimized geometry of ReH[TPC]_2_: (a) side and top views; (b) selected distances (Å).

To obtain experimental support for the DFT-derived structure, Re L_3_-edge XAS and extended X-ray absorption fine structure (EXAFS) measurements were performed on ReH[TPC]_2_ (several XAS studies of metalloporphyrins and metallocorroles have been reported in recent years^33−42^). The Re L_3_-edge absorption of ReH[TPC]_2_ was found to be blue-shifted by 2.5 eV relative to the rhenium foil, as assessed by the first derivatives of the absorption features, consistent with the significantly oxidized state of the metal in the complex (Figure S4). The nonphase-shift-corrected EXAFS data (inset), the corresponding Fourier transform, and the best fit are presented in Figure 4. Two qualitative observations may be made from the Fourier transform data: (a) the first shell is split into multiple “subshells” and (b) single- and multiple-scattering contributions from the corrole rings dominate in the ∼2.8–3.2 Å region. FEFF fits to the data reveal a seven-coordinate first shell with 1 Re–N ∼ 1.86 Å, 2 Re–N ∼ 2.37 Å, and 4 Re–N ∼ 2.55 Å (Table 1). The peaks at and below 1 Å in the Fourier transform are artifacts of the background subtraction process in which the normalized XAS data are splined to minimize oscillatory components with periods corresponding to unphysically short metal–ligand bond distances and do not indicate rhenium–ligand backscattering interactions. The longer distance Re–N paths are correlated with one another and with the Re–C single-scattering contributions from the corrole ligand (Table 1). The Fourier transform intensity in the range *R*′ ∼ 3.2–5.0 Å has multiple contributions from the two corroles. In the fit presented here (Figure 4), this long-range intensity was simulated using two multiple-scattering paths from the corrole (Re–C–N) ring. That said, these paths account for less than 10% of the overall intensity and do not impact the first-shell distances reported in Table 1. Attempts to model the first shell with a total of 8 Re–N distances (split over two or three-shells) resulted in statistically worse fits. The results suggest that ReH[TPC]_2_ is best described as having a heterogeneous first shell with a total of 7 Re–N interactions.

**Table 1:** EXAFS Least-Squares Fitting Results for ReH[TPC]_2_

path/shell	*R* (Å)^a^	σ^2^ (Å^2^)^b^	Δ*E*_0_ (eV)	*F*^c^
1 Re–N	1.86	247	–3.40	0.09
2 Re–N	2.37	425		
4 Re–N	2.55	328		
5 Re–C	2.79	637		
10 Re–C–N	3.17	462		
24 Re–C–N	4.93	1475		
24 Re–C–N	5.16	1121		

a The estimated standard deviations for the distances are on the order of ±0.02 Å.

b The σ^2^ values are multiplied by 10^5^.

c The error is given by ∑[(χ_obsd_ – χ_calcd_)^2^*k*^6^]/∑[(χ_obsd_)^2^*k*^6^]. The *S*_0_^2^ factor was set to 1.

**Figure 4:**
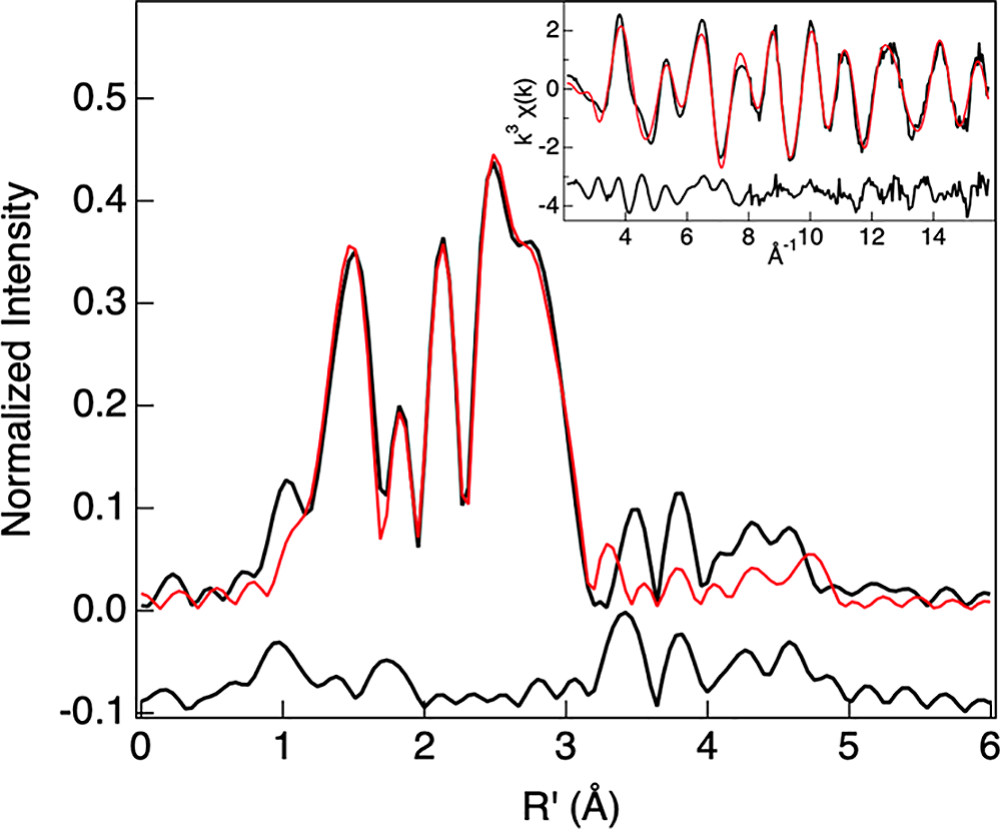
Nonphase-shift-corrected Fourier transforms of the Re L_3_-edge EXAFS data for ReH[TPC]_2_: data (black); fit (red). The inset shows the EXAFS comparison.

In summary, the high-temperature interaction of Re_2_(CO)_10_ with three free-base *meso*-triarylcorroles has led to the isolation of neutral rhenium corrole sandwich compounds with the molecular formula ReH[T*p*XPC]_2_, a heretofore unprecedented coordination motif in the field of rhenium–porphyrinoid interactions.^1,2^ DFT calculations and Re L_3_-edge EXAFS studies support a seven-coordinate rhenium center and one loosely interacting NH group. The extra hydrogen can be removed by a base, resulting in dramatically sharper UV–vis spectra consistent with *C*_2_-symmetric {Re[T*p*XPC]_2_}^−^ anions. Structural analyses of both the neutral and anionic forms remain a key goal for the future.

## Data Availability

All data generated or analyzed in this study are included in this published article and its Supporting Information.

## Supporting Information Available

Synthetic protocols, HR-ESI-MS and XAS spectra, and DFT-optimized Cartesian coordinates (PDF)
