# Supplementary material for: Rhenium Biscorrole Sandwich Compounds: XAS Evidence for a New Coordination Motif
Source: Inorg Chem. 2023 May 23;62(22):8467–71. doi: 10.1021/acs.inorgchem.3c00632 (PMC10245377; doi:10.1021/acs.inorgchem.3c00632)
Supplement: Supplementary file 1 — ic3c00632_si_001.pdf [file ic3c00632_si_001.pdf]

Supporting Information

Rhenium Biscorrole Sandwich Compounds: XAS Evidence for A New Coordination Motif

Abraham B. Alemayehu,[a] Macon Jedediah Abernathy,[b] Jeanet Conradie,[a,c] Ritimukta Sarangi*,[b] and Abhik Ghosh*,[a]

[a] Department of Chemistry, University of Tromsø, N-9037 Tromsø, Norway;
[b] Stanford Synchrotron Radiation Lightsource, SLAC National Accelerator Laboratory, Stanford University, Menlo Park, California 94025, USA;
[c] Department of Chemistry, University of the Free State, P.O. Box 339, Bloemfontein 9300, Republic of South Africa

Table of contents

A. Synthetic protocol S2
B. Electrospray ionization mass spectra S3
C. XAS methods S5
D. DFT optimized coordinates S7

A. Synthesis and characterization

Materials. Free-base *meso*-triarylcorroles were synthesized according to a literature procedure (Koszarna, B.; Gryko, D. T. Efficient Synthesis of *meso*-Substituted Corroles in a H_2O-MeOH Mixture. *J. Org. Chem.* **2006**, *71*, 3707-3717.). All other reagents were purchased from Merck and used as received, except pyrrole which was distilled and stored in the freezer. Silica gel 60 (0.04-0.063 mm particle size, 230-400 mesh, Merck) was employed for flash chromatography. Silica gel 60 preparative thin-layer chromatographic plates (20 cm x 20 cm x 0.5 mm, Merck) were used for final purification of all compounds.

Instrumental methods. UV-visible spectra were recorded on an HP 8454 spectrophotometer. ^{1}H NMR spectra were recorded on a 400 MHz Bruker Avance III HD spectrometer equipped with a 5 mm BB/1H SmartProbe and referenced to either residual CH_2Cl_2 at 5.32 ppm or residual $CHCl_3$ at 7.26 ppm. High-resolution electrospray-ionization mass spectra were recorded on an Orbitrap Exploris 120 spectrometer using methanolic solutions.

General synthetic method. A 100-mL two-necked flask equipped with a magnetic stirrer was charged with free-base corrole (H_3[T*p*XPC], 0.162 mmol), $Re_2(CO)_{10}$ (105.8 mg, 0.162 mmol), K_2CO_3 (100 mg), and 1,2-dichlorobenzene (10 mL). The mixture was degassed with a stream of argon for 10 min, heated to ~235 °C, and maintained at that temperature overnight. Upon colling to room temperature, the reaction mixture was loaded onto a silica gel column with heptane as the mobile phase. 1,2-Dichlorobenzene was removed first by eluting with *n*-heptane. The ReO corrole, the first red band, was the next to elute with 3:1 v/v heptane/dichloromethane. A greenish band containing the Re corrole eluted next with 3:2 v/v heptane/dichloromethane. Finally, a brown band containing the Re biscorrole sandwich eluted with pure dichloromethane. Yields and spectroscopic details are as follows.

ReH[TPC]$_2$. Yield 20 mg (20%). UV-vis (CH_2Cl_2) λ_{max} [nm, ε x 10^{-4} ($M^{-1}cm^{-1}$)]: 362 (4.6), 517 (1.54), 585 (1.03); UV-vis (toluene) λ_{max} [nm, ε x 10^{-4} ($M^{-1}cm^{-1}$)]: 363 (3.95), 431 (2.61), 517 (1.19), 586 (0.81). MS (ESI): [M^+] = 1234.3491 (expt), 1234.3482 (calcd for $C_{74}H_{47}N_8Re$).

{Re[TPC]$_2$}$^-$. UV-vis (toluene/DBU) λ_{max} [nm, ε x 10^{-4} ($M^{-1}cm^{-1}$)]: 367 (3.18), 437 (3.86), 552 (0.74), 585 (0.86). MS (ESI): [M^-] = 1233.3423 (expt), 1233.3403 (calcd for $C_{74}H_{46}N_8Re$).

ReH[T*p*CH$_3$PC]$_2$. Yield 23 mg (21.53%). UV-vis (CH_2Cl_2) λ_{max} [nm, ε x 10^{-4} ($M^{-1}cm^{-1}$)]: 366 (3.83), 520 (1.20), 586 (0.67); UV-vis (toluene) λ_{max} [nm, ε x 10^{-4} ($M^{-1}cm^{-1}$)]: 370 (3.68), 519 (1.08), 584 (0.58). MS (ESI): [M^+] = 1318.4462 (expt), 1318.4422 (calcd for $C_{80}H_{59}N_8Re$).

Re[T*p*CH$_3$PC]$_2$}$^-$. UV-vis (toluene/DBU) λ_{max} [nm, ε x 10^{-4} ($M^{-1}cm^{-1}$)]: 369 (3.11), 439 (3.45), 552 (0.70), 585 (0.78). MS (ESI): [M^-] = 1233.3423 (expt), 1233.3403 (calcd for $C_{80}H_{58}N_8Re$).

ReH[T*p*OCH$_3$PC]$_2$. Yield 27 mg (23.56%). UV-vis (CH_2Cl_2) λ_{max} [nm, ε x 10^{-4} ($M^{-1}cm^{-1}$)]: 403 (4.32), 509 (1.57), 583 (0.98); UV-vis (toluene) λ_{max} [nm, ε x 10^{-4} ($M^{-1}cm^{-1}$)]: 399 (3.46), 520 (1.26), 583 (0.74). MS (ESI): [M^+] = 1414.4166 (expt), 1314.4117 (calcd for $C_{80}H_{59}N_8O_6Re$).

Re[T*p*OCH$_3$PC]$_2$}$^-$. UV-vis (toluene/DBU) λ_{max} [nm, ε x 10^{-4} ($M^{-1}cm^{-1}$)]: 374 (2.80), 439 (2.70), 592 (0.62). MS (ESI): [M^-] = 1413.4055 (expt), 1413.4038 (calcd for $C_{80}H_{58}N_8O_6Re$).

B. Electrospray ionization mass spectra

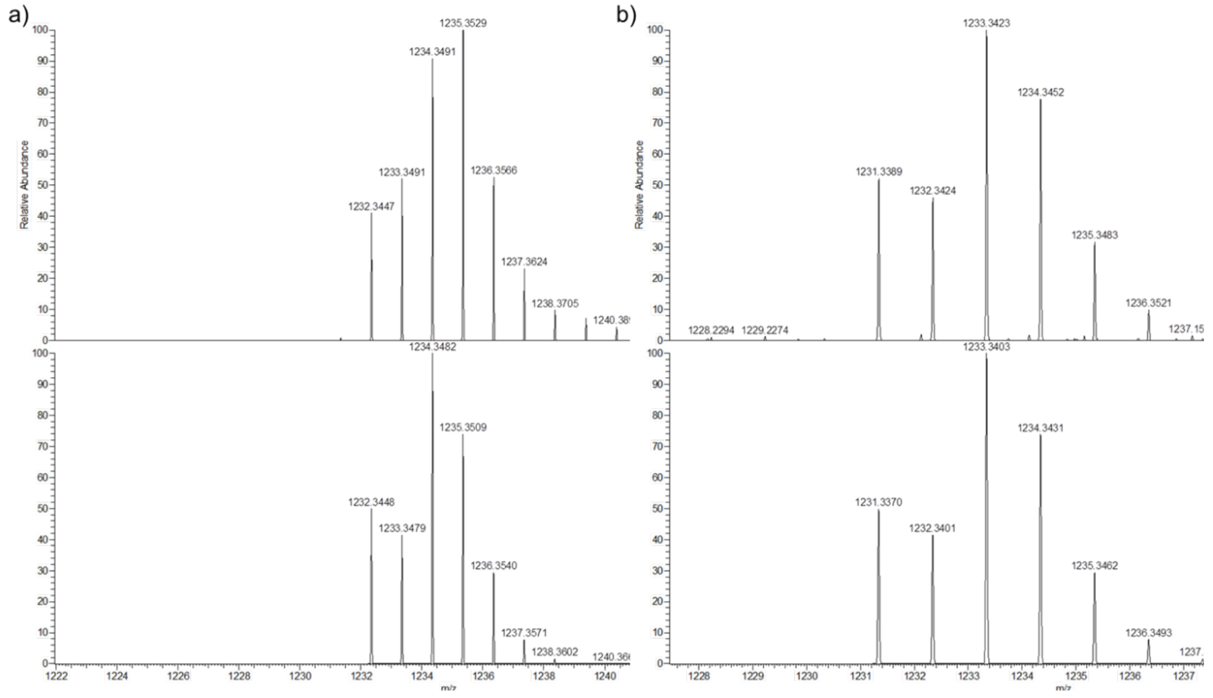

Figure S1. HRMS molecular ion peaks of ReH[TPC]$_2$: (a) without DBU for $C_{74}H_{47}N_8Re$ in positive mode and (b) with DBU for $C_{74}H_{46}N_8Re$ in negative mode.

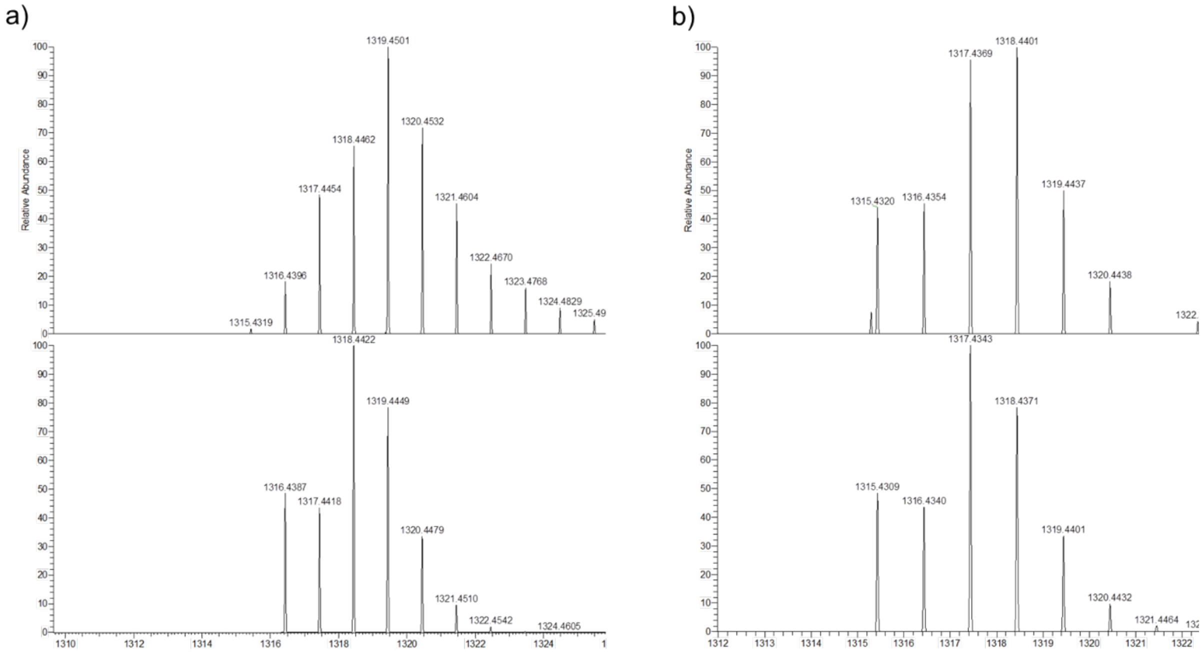

Figure S2. HRMS molecular ion peaks of ReH[T*p*CH_3PC]$_2$: (a) without DBU for $C_{80}H_{59}N_8Re$ in positive mode and (b) in DBU for $C_{80}H_{58}N_8Re$ in negative mode.

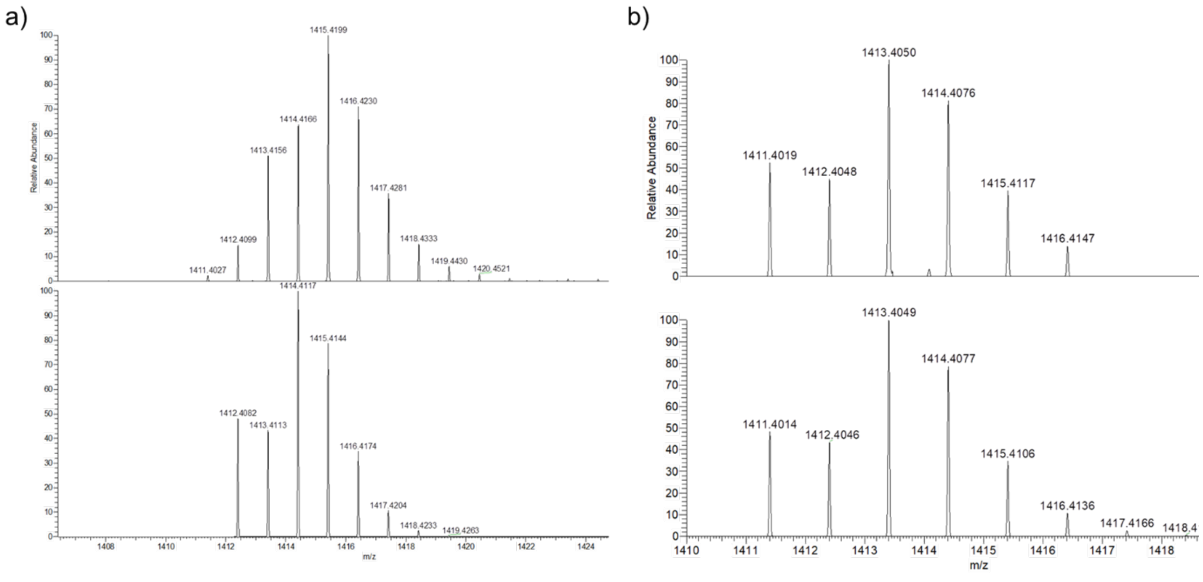

Figure S3. HRMS molecular ion peaks of Re[T*p*OCH_3PC]$_2$: (a) without DBU for $C_{80}H_{59}N_8Re$ in positive mode and (b) with DBU for $C_{80}H_{58}N_8Re$ in negative mode.

C. XAS methods

Rhenium L_3-edge X-ray absorption spectra were collected at the Stanford Synchrotron Radiation Lightsource under standard ring conditions of 3 GeV and 500 mA. Spectra were collected at beamline 9-3 and a 16-pole, 2.0 T wiggler side-station. Energy selection was provided by a monochromator equipped with a Si(220) crystal set that was oriented to ϕ = 90°. Higher order harmonics were rejected from the incoming photon beam using a Rh-coated mirror. A sample of ReH[TPC]$_2$ was diluted with boron nitride to 3.5% Re by mass prior to data collection. The diluted sample was mounted as a powder and maintained at 10 K during data collection using an Oxford Instruments CF1208 liquid helium cryostat. Unwanted X-ray scattering from the sample was minimized with a Zn filter with a thickness of 6 absorption lengths and a set of Soller slits placed between the sample cryostat and the detector. Fluorescence spectra were collected with a PIPS detector equipped with a Mylar cover to exclude unwanted light. Spectra were collected out to a k = 16 Å^{-1}, with two scans taken on two different spots on the sample. No photo damage was observed when comparing the first and second scans collected on each spot. An Re foil was placed between two N_2-filled ion chambers in line with the sample for simultaneous collection of reference spectra, in which the first derivative was assigned to 10535 eV.

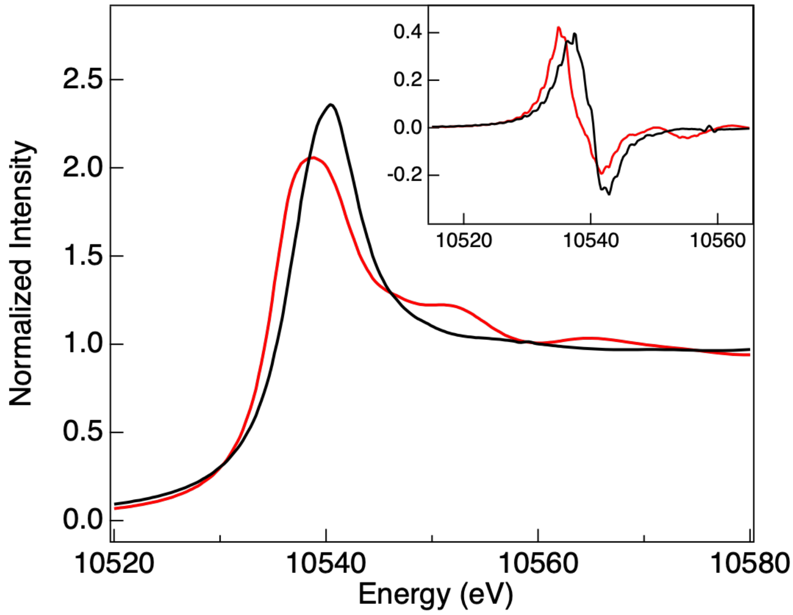

Figure S4. (a) Normalized Re L_3-edge XAS data for ReH[TPC]$_2$ (black line) and Re foil (red line). Inset: First derivative of the normalized XANES highlighting the blueshift of the sample's edge relative to Re foil.

The spectra were calibrated against reference foil spectra, and normalized with Athena[1] (v 9.26, Windows) using a linear function to model the pre-edge region and a third order polynomial to model the post-edge region. The four normalized spectra were then averaged and exported to Pyspline,[2] where a five-region spline of orders 2, 3, 3, 3, 3 was applied to the data to minimize frequency contributions occurring below 1 Å in the Fourier-transformed data. The k^3-weighted data was fit in EXAFSPAK[3] using theoretical EXAFS signals generated with FEFF[4] using a DFT optimized geometry. The bond distance and bond disorder (σ^2) were varied for each scattering path present in the fit, while the energy parameter E_0 was varied with each fit but set to a common value for each scattering contribution within a given fit, and coordination numbers were varied systematically between fits. The amplitude reduction factor S_0^2 was fixed to 0.9.

(1) Ravel, B.; Newville, M. ATHENA, ARTEMIS, HEPHAESTUS: Data Analysis for X-Ray Absorption Spectroscopy Using IFEFFIT. *J. Synchrotron Radiat.* **2005**, *12* (4), 537–541. https://doi.org/10.1107/S0909049505012719.

(2) Tenderholt, A.; Hedman, B.; Hodgson, K. O. PySpline: A Modern, Cross-Platform Program for the Processing of Raw Averaged XAS Edge and EXAFS Data. *AIP Conf. Proc.* **2007**, *882*, 105–107. https://doi.org/10.1063/1.2644442.

(3) George, G. N.; Pickering, I. J. EXAFSPAK and EDG-FIT. *Stanf. Synchrotron Radiat. Lab. Stanf. Linear Accel. Cent. Stanf. CA*.

(4) Ankudinov, A. L.; Rehr, J. J. Relativistic Calculations of Spin-Dependent x-Ray-Absorption Spectra. *Phys. Rev. B* **1997**, *56* (4), R1712–R1716. https://doi.org/10.1103/PhysRevB.56.R1712.

D. All-electron OLYP-D3/ZORA-STO-TZ2P SR optimized Cartesian coordinates (Å)

Re	-0.330822000	-0.206084000	0.272891000
C	0.340866000	3.481646000	-1.243474000
C	0.372202000	4.962808000	-1.155864000
C	0.445993000	2.532540000	1.803098000
C	0.477943000	-2.275469000	-1.945829000
C	0.550777000	-4.282996000	1.235342000
C	0.573550000	7.767206000	-0.902981000
C	0.945287000	-2.923985000	1.067153000
C	1.144037000	5.718607000	-2.055301000
C	1.250275000	7.101347000	-1.926420000
C	1.386440000	-3.133391000	-2.619826000
C	1.577861000	2.850154000	-1.452828000
C	1.824703000	2.373634000	1.730847000
C	2.253960000	-2.437301000	1.074544000
C	2.360502000	1.128971000	1.387506000
C	2.429994000	-1.247173000	-1.896994000
C	2.552991000	-1.074249000	1.120847000
C	2.568834000	4.762463000	1.648792000
C	2.607607000	-2.505332000	-2.573230000
C	2.697844000	3.478593000	2.194480000
C	2.912285000	3.348525000	-1.318804000
C	3.015533000	1.126162000	-1.760554000
C	3.362991000	-3.425848000	1.122330000
C	3.365036000	5.812756000	2.097793000
C	3.395433000	-0.218082000	-1.907859000
C	3.487883000	-4.382385000	0.105348000
C	3.624233000	3.282624000	3.230519000
C	3.750414000	0.806879000	1.439521000
C	3.784626000	2.298706000	-1.505249000
C	3.869796000	-0.539674000	1.279934000
C	4.301860000	5.601260000	3.110506000
C	4.305960000	-3.423723000	2.159896000
C	4.422401000	4.332315000	3.680421000
C	4.550723000	-5.281576000	0.100191000
C	4.812601000	-0.558797000	-2.116005000
C	5.370655000	-4.322615000	2.154950000
C	5.373430000	-1.679909000	-1.478383000
C	5.502662000	-5.249724000	1.119876000
C	5.644132000	0.214297000	-2.945884000
C	6.713551000	-2.009905000	-1.653148000
C	6.986802000	-0.112617000	-3.116875000
C	7.530781000	-1.224456000	-2.469100000
C	-0.223028000	7.034133000	-0.021644000
C	-0.288391000	3.584425000	2.430190000
C	-0.324117000	5.652703000	-0.149049000
C	-0.818249000	-4.310994000	1.276074000
C	-0.911142000	2.819956000	-1.177015000
C	-0.913316000	-2.284202000	-1.830630000
C	-1.286217000	-2.971039000	1.153744000

C	-1.613676000	3.207804000	2.469760000
C	-1.686204000	1.924963000	1.878022000
C	-1.957959000	-2.951992000	-2.516685000
C	-2.128182000	3.562076000	-1.348960000
C	-2.611881000	1.365607000	-1.271287000
C	-2.629872000	-2.589123000	1.303494000
C	-2.642666000	0.919892000	1.763252000
C	-2.736331000	-1.044496000	-1.567237000
C	-2.995480000	-1.258593000	1.455952000
C	-3.097922000	-2.185593000	-2.348452000
C	-3.162846000	2.676898000	-1.383227000
C	-3.371844000	0.197707000	-1.476836000
C	-3.667968000	-3.644656000	1.424761000
C	-3.953038000	-4.465862000	0.326343000
C	-3.970485000	0.661696000	2.177008000
C	-4.199517000	-0.685999000	1.963787000
C	-4.380263000	-3.830174000	2.616629000
C	-4.806827000	0.228058000	-1.839843000
C	-4.943593000	-5.442459000	0.414108000
C	-5.303994000	1.033841000	-2.876706000
C	-5.367103000	-4.811281000	2.704894000
C	-5.655253000	-5.617628000	1.602230000
C	-5.689880000	-0.665595000	-1.211589000
C	-6.646066000	0.974935000	-3.245986000
C	-7.030530000	-0.728389000	-1.583328000
C	-7.517925000	0.098197000	-2.597194000
H	0.159141000	4.479957000	2.832135000
H	0.659025000	8.846322000	-0.799421000
H	0.926534000	0.888178000	-1.873592000
H	1.136220000	-4.075672000	-3.089014000
H	1.234201000	-5.110680000	1.344983000
H	1.663324000	5.208048000	-2.859426000
H	1.841166000	4.930822000	0.868846000
H	1.857720000	7.661971000	-2.633407000
H	2.761079000	-4.384655000	-0.696256000
H	3.165192000	4.360269000	-1.052186000
H	3.250630000	6.798349000	1.652751000
H	3.531876000	-2.819523000	-3.034664000
H	3.703150000	2.303357000	3.691194000
H	4.200156000	-2.707715000	2.968402000
H	4.537876000	1.528056000	1.584998000
H	4.641126000	-6.002218000	-0.709409000
H	4.747659000	-2.285441000	-0.837998000
H	4.776160000	-1.118801000	1.258023000
H	4.858622000	2.316526000	-1.409530000
H	4.925623000	6.419983000	3.461104000
H	5.132179000	4.161090000	4.486581000
H	5.220548000	1.062500000	-3.473830000
H	6.096134000	-4.302526000	2.964894000
H	6.336805000	-5.947351000	1.112815000
H	7.120377000	-2.880221000	-1.143455000

H	7.609511000	0.495208000	-3.769751000
H	8.579241000	-1.478871000	-2.604737000
H	-0.758016000	7.539664000	0.779460000
H	-0.917808000	5.083387000	0.552298000
H	-1.461432000	-5.165430000	1.423734000
H	-1.840901000	-3.835755000	-3.129144000
H	-2.177827000	4.628118000	-1.502852000
H	-2.444946000	3.747829000	2.902639000
H	-3.401543000	-4.316422000	-0.594743000
H	-4.067376000	-2.330442000	-2.801300000
H	-4.150139000	-3.204071000	3.473372000
H	-4.209485000	2.895617000	-1.522357000
H	-4.622308000	1.685833000	-3.412488000
H	-4.652844000	1.384455000	2.603390000
H	-5.097447000	-1.241810000	2.184583000
H	-5.162752000	-6.066217000	-0.449736000
H	-5.308223000	-1.310208000	-0.429510000
H	-5.909102000	-4.948911000	3.637725000
H	-6.427460000	-6.380408000	1.669963000
H	-7.008832000	1.603668000	-4.056180000
H	-7.696636000	-1.425573000	-1.079604000
H	-8.564689000	0.051472000	-2.888409000
N	1.108859000	-1.144270000	-1.485074000
N	1.623302000	-0.024822000	1.097134000
N	1.702399000	1.524484000	-1.756538000
N	-0.184705000	-2.110629000	1.007280000
N	-0.434754000	1.516700000	1.463702000
N	-1.215804000	1.453331000	-1.073141000
N	-1.426167000	-1.181013000	-1.186418000
N	-2.076048000	-0.230707000	1.273203000
